# Recent Advances in the Reactions of Cyclic Carbynes

**DOI:** 10.3390/molecules25215050

**Published:** 2020-10-30

**Authors:** Qian Su, Jipeng Ding, Zhihui Du, Yunrong Lai, Hongzuo Li, Ming-An Ouyang, Liyan Song, Ran Lin

**Affiliations:** Key Laboratory of Biopesticide and Chemical Biology (Ministry of Education), College of Plant Protection, Fujian Agriculture and Forestry University, Fuzhou 350002, China; fafusuqian@163.com (Q.S.); mrshujian@163.com (J.D.); duzhihui010@126.com (Z.D.); laiyunrong214@163.com (Y.L.); lhz8290@163.com (H.L.); minganouyang@163.com (M.-A.O.)

**Keywords:** cyclic carbynes, reactivities, metallabenzynes, metallapentalynes

## Abstract

The acyclic organic alkynes and carbyne bonds exhibit linear shapes. Metallabenzynes and metallapentalynes are six- or five-membered metallacycles containing carbynes, whose carbine-carbon bond angles are less than 180°. Such distortion results in considerable ring strain, resulting in the unprecedented reactivity compared with acyclic carbynes. Meanwhile, the aromaticity of these metallacycles would stabilize the ring system. The fascinating combination of ring strain and aromaticity would lead to interesting reactivities. This mini review summarized recent findings on the reactivity of the metal–carbon triple bonds and the aromatic ring system. In the case of metallabenzynes, aromaticity would prevail over ring strain. The reactions are similar to those of organic aromatics, especially in electrophilic reactions. Meanwhile, fragmentation of metallacarbynes might be observed via migratory insertion if the aromaticity of metallacarbynes is strongly affected. In the case of metallapentalynes, the extremely small bond angle would result in high reactivity of the carbyne moiety, which would undergo typical reactions for organic alkynes, including interaction with coinage metal complexes, electrophilic reactions, nucleophilic reactions and cycloaddition reactions, whereas the strong aromaticity ensured the integrity of the bicyclic framework of metallapentalynes throughout all reported reaction conditions.

## 1. Introduction

**Figure 1:**
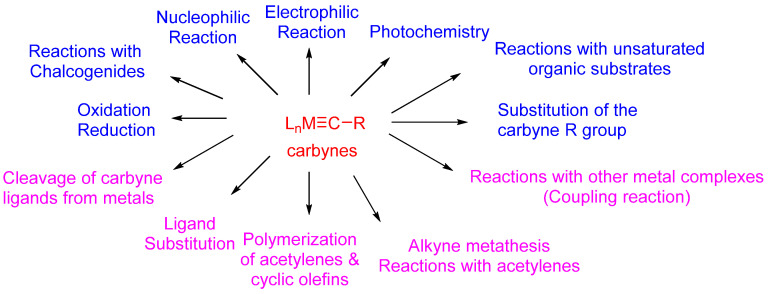
Reactions of acyclic carbynes.

The alkyne (carbon-carbon triple bond), which is a fundamental functional group in organic chemistry, is involved in a large number of reactions in organic chemistry [1,2,3,4,5]. Carbyne complexes, i.e., transition metal complexes with metal-carbon triple bonds, can be described as analogs of alkynes, whereas a transition metal replaces one of the sp carbons. They have attracted considerable attention because of their remarkable features and their significance as catalysts or reagents for various types of organic transformations [5,6,7,8,9,10,11]. Part of the reactivities of carbynes paralleled that of their organic parents, such as nucleophilic reactions, electrophilic reactions, photochemistry, oxidation and reduction, reactions with chalcogenides, reactions with unsaturated organic substrates (cycloadditions) and substitution on the α carbon (Figure 1). However, the incorporation of metal into the triple would lead to fascinating properties, which were quite different from organic alkynes. For example, late-transition-metal carbyne species were applied as catalysts in alkyne metathesis or alkyne polymerization reactions. Carbynes might be coupled or reactive to other metal complexes to afford bi- or multi-metallic complexes. Ligand substitution is one of the fundamental reactions in organometallic chemistry, which is certainly involved in the reactions of carbynes. In addition, carbyne ligands might be cleaved from metal centers to give various organic products (Figure 1).

**Figure 2:**
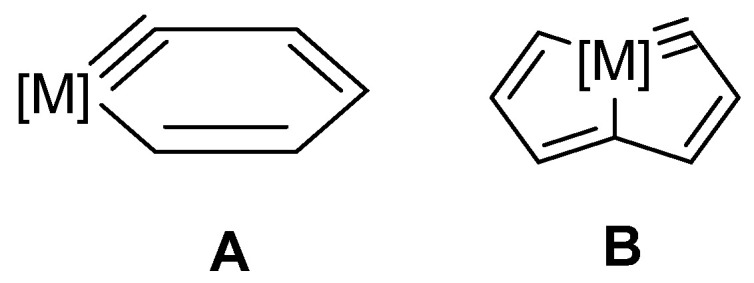
Strained metallacycles with carbyne bond.

The alkyne moiety is normally linear due to the sp hybridization of acetylenic carbon. Ring strain would be raised from the introduction of alkyne moiety into an organic cycle. As a result, limited insights could be gained into the reactivity of organic cyclic alkynes due to the instability caused by the high ring tension [12,13]. The incorporation of a metal center into the acetylenic carbon would stabilize the strained cyclic carbon cycles containing alkynes. The cyclic carbynes obtained until now could be classified as six-membered metallabenzynes [14,15,16,17,18,19,20,21] **A** and five-membered metallapentalynes [22,23,24] **B** (Figure 2). In addition, the first metallapyridyne [25] was documented by Xia and coworkers, providing one precious example of cyclic carbynes.

**Figure 3:**
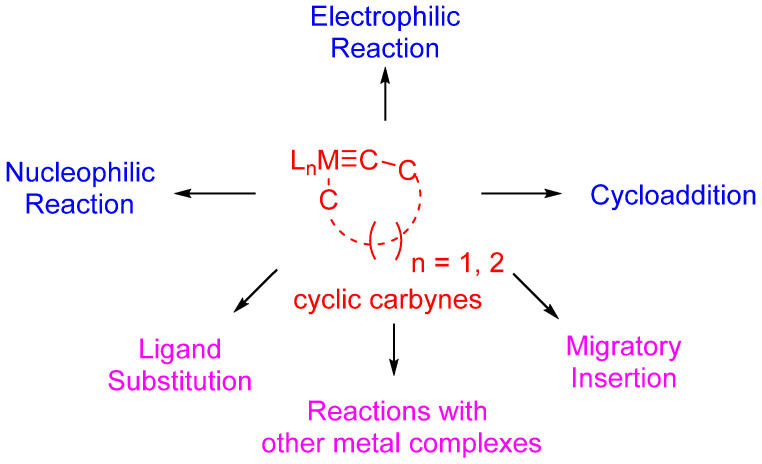
Reactions of cyclic carbynes.

The difference between acyclic carbynes and cyclic carbynes might be due to the ring strain. The chemical properties of cyclic carbynes are summarized in Figure 3. Among the limited examples, the reaction types of cyclic carbynes are similar to those of acyclic carbynes except that in most cases the “migratory insertion” [26] would not stop at the carbene complexes and lead to the transformation of aromatic metallacycles into cyclopentadienes (Cp) complexes [27].

Carbynes are usually recognized as important intermediates in many transition-metal-catalyzed organic reactions [28,29]. The thorough investigation of the reactivities and properties might provide a new perspective for the application of late-transition-metal carbyne species as prosperous catalysts in various organic reactions. The chemistry of metallabenzynes has been fully illustrated and nicely reviewed [14,15,16,17,18,19,20,21]. However, there is no review paper covering the entirety of cyclic carbynes (metallabenzynes and metallapentalynes). This account deals with them together for the first time and the scope has been limited to the reactivities of metallabenzynes and metallapentalynes, seeking to provide a general picture of reactivity of the metal–carbon triple bonds or the entire aromatic system.

## 2. Reactivity of Six-Membered Metallabenzynes

**Figure 4:**
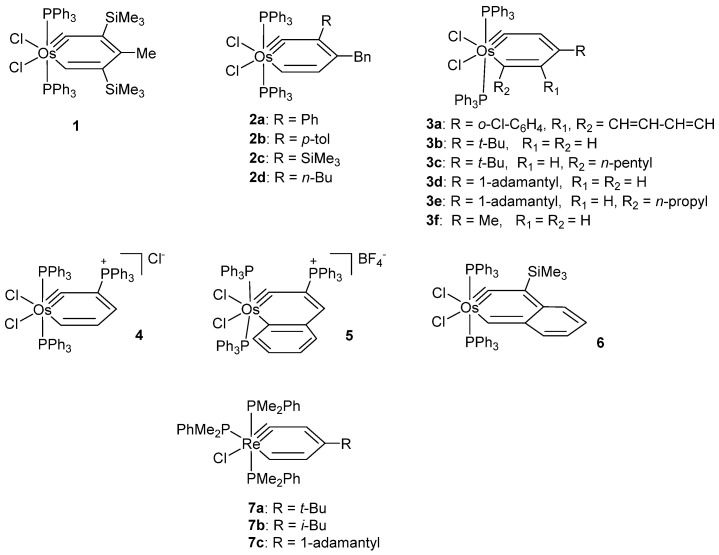
Metallabenzynes reported to date.

Metallabenzynes have been recognized as transition metal analogs of benzyne in which a C atom is replaced by an isolobal transition metal fragment. The chemical structure of metallabenzynes exhibits aromaticity, which would lead to the similar reactions occurring in their organic counterparts. On the other hand, the carbyne moiety (M≡C, metal-carbon triple bond) is reactive to several reagents and might involve regular reactions that normal acyclic carbynes would experience. The first metallabenzyne **1** was secured by Jia [30] and co-workers in 2001. Then, many interesting metallabenzynes were produced and well-characterized. (Figure 4) [31,32,33,34,35,36,37,38,39,40] Most of the metallabenzynes are osmabenzynes (**1**–**6**) [30,31,32,33,34,35,36,37,38]. Rhenabenzynes (**7**), which are present in very limited amounts, were reported by Jia’s group [39,40]. Moreover, only osmabenzynes and rhenabenzynes have been discovered to date.

**Scheme 1:**
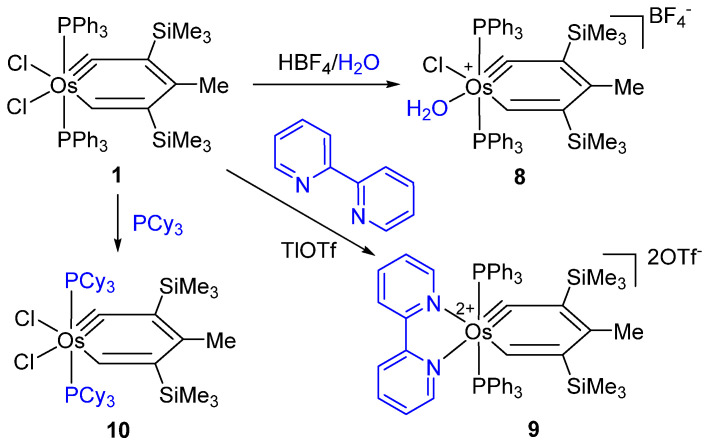
Ligand exchange reactions of osmabenzyne **1**.

The cationic osmabenzyne **8** was formed by treatment of metallabenzyne **1** [30] with two equivalent HBF_4_ in wet dichloromethane. The anionic chloride ligand was replaced by the neutral water with the aid of Bronsted acid [41]. Similarly, the dicationic osmabenzyne **9** was prepared by the replacement of chloride ligands in **1** with 2,2′-bipyridine (bipy) in the presence of thallium triflate (TlOTf) [42]. The exchange of phosphine ligands was much easier, and no reagents and catalysts were needed. Excess PCy_3_ in refluxing benzene would simply kick out the triphenylphospine and result in neutral osmabenzyne **10** [43]. The transformations are depicted in Scheme 1.

### 2.1. Electrophilic Substitution Reaction

**Scheme 2:**
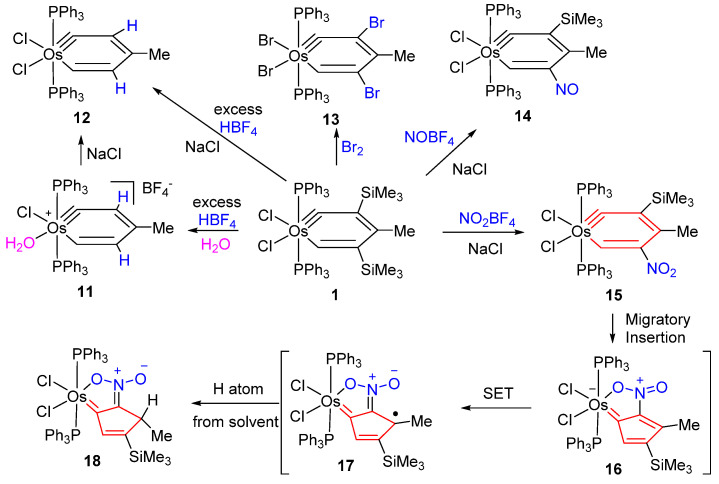
Electrophilic reactions of osmabenzyne **1**.

Trimethylsilyl group (TMS) was usually recognized as the “equivalent” of cationic hydrogen and labile to electrophiles in organic reactions. In the presence of excess tetrafluoroboric acid and water, metallabenzyne **1 [30]** was converted to cationic metallabenzyne **11** via electrophilic desilylation and subsequent ligand exchange. Sodium chloride would provide anionic chloride ion to avoid ligand exchange to facilitate the formation of neutral desilylated osmabenzyne **12**. Treatment of isolated **11** with NaCl also gave **12**. Bromination of metallabenzyne **1** with excess elemental bromine readily provides brominated osmabenzyne **13**, in which both the Me_3_Si and Cl in **1** have been replaced by Br [41]. Nitrosation of metallabenzyne **1** was carried out in the presence of excess nitrosonium salt (NOBF_4_) and sodium chloride at low temperature to furnish osmabenzyne **14** in which the SiMe_3_ substituent at C4 was replaced by NO [44]. Nitration of metallabenzyne **1** took place at C4 to afford osmabenzyne **15** under similar reaction conditions except that nitronium salt (NO_2_BF_4_) was applied. Surprisingly, the biclyclic species **18** was isolated together with osmabenzyne **15**. A plausible mechanism was rationalized. Initially, electrophilic substitution of NO_2_^+^ produced osmabenzyne **15**, which might undergo migratory insertion reaction and coordination of anionic oxygen atom to give intermediate **16**. Subsequent single electron transfer generated the radical **17**, which would abstract a hydrogen atom from solvent to give the osmium complex **18** [44]. The reactions of osmabenzyne **1** is summarized in Scheme 2.

**Scheme 3:**
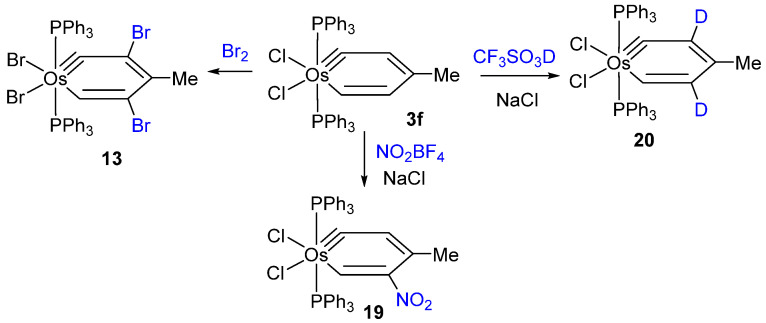
Electrophilic reactions of osmabenzyne **3f**.

It is well known that C-SiMe_3_ bonds on an aromatic ring are more reactive than C-H bonds towards electrophiles. In order to further investigate the reactivity of metallabenzynes without SiMe_3_ group, osmabenzyne **3f** was subjected to electrophilic conditions (Scheme 3). Bromination and nitration were carried out under similar conditions and provided the identical brominated product **13** and mononitrated osmabenzyne **19,** respectively [44]. Treatment of osmabenzyne **3f** with CF_3_SO_2_D in the presence of sodium chloride produced 3,5-dideuterated osmabenzyne **20** [41], which indicates that the carbons of Os*C* or Os*CH* were not attacked by the acid under the protonation condition.

**Scheme 4:**
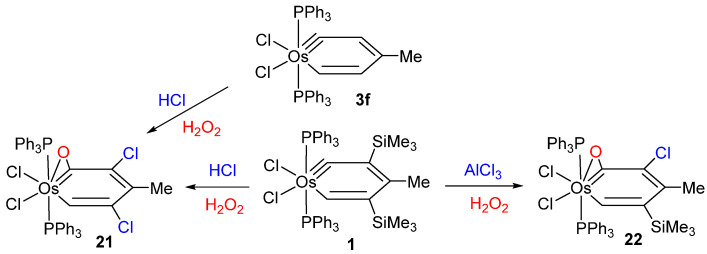
Chlorination reactions of osmabenzynes **1** and **3f**.

Chlorination of osmabenzyne **1** and **3f** with excess HCl/H_2_O_2_ furnished the same 3,5-dichlorinated product **21**, while C2 monochlorinated product **22** could be obtained when the ratio of AlCl_3_/H_2_O_2_ was carefully controlled [44]. Noteworthy is that the carbyne moiety was attacked by oxygen atoms in the presence of hydrogen peroxide to afford metalla-oxirene fragments in the chlorination reactions (Scheme 4).

The electrophilic substitution reactions of metallabenzynes resemble the organic arenes and the metal-carbon triple bonds stayed intact under most electrophilic conditions except when highly oxidative H_2_O_2_ was applied. These phenomena demonstrate the aromatic properties of metallabenzynes.

### 2.2. Nucleophilic Reaction

**Scheme 5:**
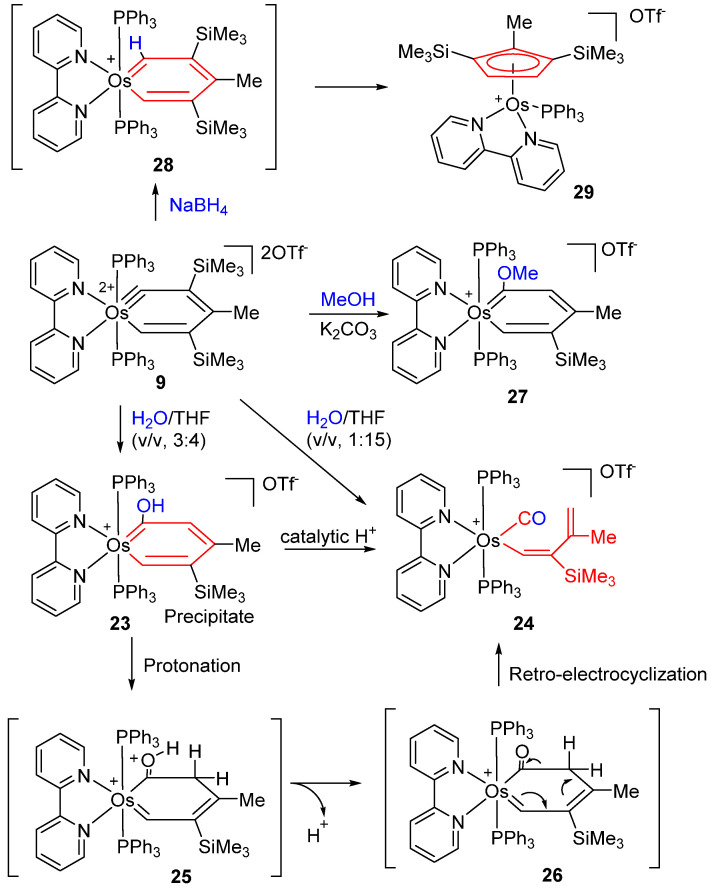
Nucleophilic reactions of osmabenzyne **9**.

The carbyne moiety in metallabenzyne could be attacked by nucleophiles. The first nucleophilic reactions of metallabenzynes were explored by Jia (Scheme 5) [42]. In order to increase the electrophilicity of metallacycle, the neutral osmabenzyne **1** was transformed to dicationic osmabenzyne **9** by ligand exchange, which was quite electrophilic and readily reacted with nucleophiles (Scheme 5). Water, a weak nucleophile, participated in the nucleophilic attack of the carbyne atom to generate metallaphenol **23**, which precipitated from the solvent system (H_2_O/THF = 3:4). Desilylation was also observed on the SiMe_3_ adjacent to carbyne carbon, which might be attributed to the higher nucleophilicity of the Si-bound carbon. The metallaphenol **23** could be transformed to acyclic complex **24** in the presence of catalytic HBF_4_. Protonation and enol-ketone transformation might give intermediate **25**, which was deprotonated to afford the ketone form **26**. A six-electron retro-electrocyclization reaction of **26** would furnish acyclic complex **24**. Moreover, osmabenzyne could be transformed to acyclic complex **24** directly if better solubility (H_2_O/THF = 1:15) and longer reaction time (two days) were provided. The combination of methanol and potassium carbonate not only provided methoxide to conduct the nucleophile addition to the carbyne carbon, but also removed the trimethylsilyl group adjacent to the carbyne carbon, resulting in the similar product **27**. The hydride provided by NaBH_4_ attacked the carbyne carbon of osmabenzyne **9** to give the metallabenzene intermediate **28**, which would undergo migratory insertion reaction and subsequent rearrangement to afford cyclopentadienyl complex **29**. The regioselectivity of nucleophilic attack is C1 (carbyne carbon) in Scheme 5.

**Scheme 6:**
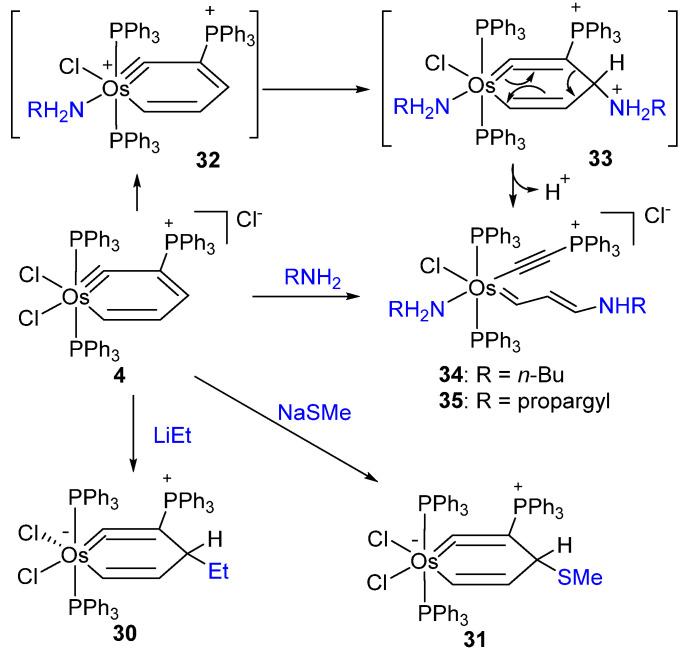
Nucleophilic reactions of osmabenzyne **4**.

Xia and coworkers reported nucleophilic addition to metallabenzynes with different regioselectivity (C3) [36]. They prepared osmabenzyne **4** and subjected it to nucleophilic addition with stronger nucleophiles, such as ethyllithium (EtLi) or sodium methanethiolate (NaSMe). Not surprisingly, the carbyne carbon (C3) was attacked to generate isoosmabenzene **30** or **31**. If primary amine was applied, it would kick out the chloride ligand to give the dicationic osmabenzyne **32**, which is more electrophilic than **4**. Hence, the nucleophilic attack of primary amine on C3 and subsequent rearrangement of isoosmabenzene **33** furnished the ring-opened product **34** or **35** (Scheme 6).

### 2.3. Migratory Insertion Reactions

**Scheme 7:**
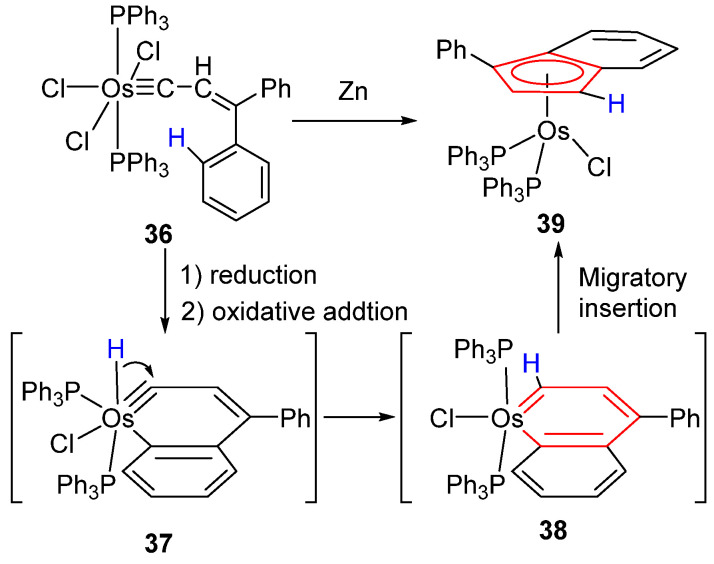
Zinc reduction of vinylcarbyne complex **36**.

Metallabenzenes and metallabenzynes usually exhibit rearrangement to the corresponding cyclopentadienyl complexes, which is the major decomposition pathway. The migratory insertion step involves reductive elimination, in which the two carbon atoms adjacent to metal undergo the coupling reaction to form cyclopentadienyl moiety. In 2007, Jia and Lin [37] discovered that reduction of complex **36** and subsequent cyclometallation gave hydrido osmabenzyne intermediate **37**, which underwent hydride shift to attack the carbyne carbon to afford metallabenzene intermediate **38**. Intermediate **38** would undergo migratory insertion reaction and subsequent rearrangement to afford cyclopentadienyl (Cp) complex **39** (Scheme 7).

**Scheme 8:**
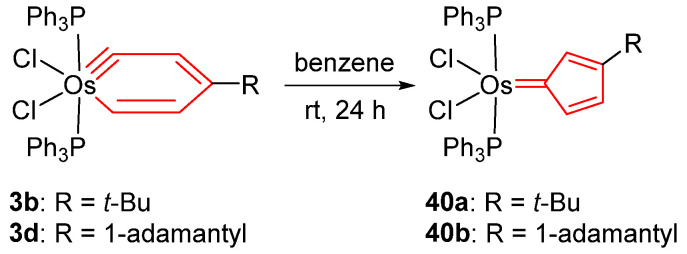
Substituent effect on migratory insertion reaction.

Metallabenzynes could also undergo migratory insertion reactions to give carbene complexes. The substituent effect was illustrated by Jia and Lin. As depicted in Scheme 8, osmabenzynes bearing bulky substituents (*t*-butyl or 1-adamantyl) *para* to the metal center would slowly convert to the corresponding osmium carbene complexes **40a** and **40b** at room temperature in solution [38]. The steric hindrance of the bulky substituents might prevent the osmium carbene complexes from further transformation [45,46].

**Scheme 9:**
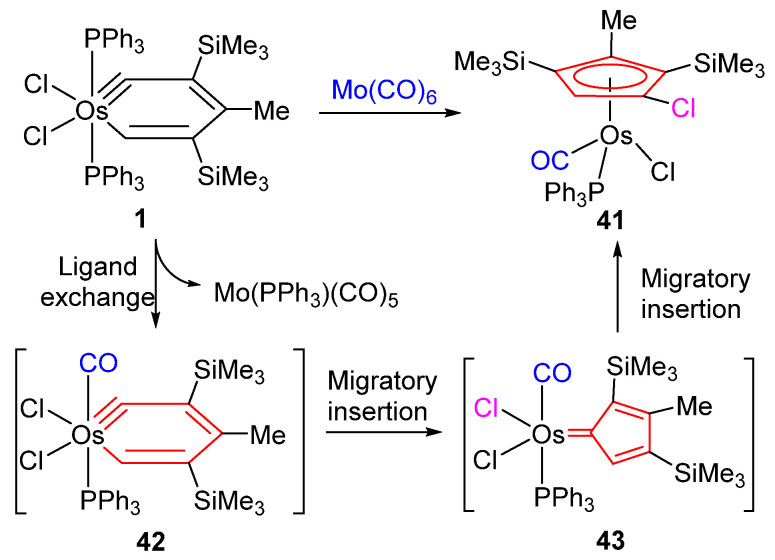
Ligand effect on migratory insertion reaction.

The ligand effect played an important role in the rearrangement of osmabenzyne **1** to the corresponding cyclopentadienyl complex **41**, which was demonstrated by Jia [43] as shown in Scheme 9. Ligand exchange reaction of stable osmabenzyne **1** with Mo(CO)_6_ would replace one phosphine ligand with carbon monoxide and deliver reactive osmabenzyne intermediate **42**, which underwent migratory insertion involving the two metal-bonded carbons to give the carbene intermediate **43**. Migratory insertion of the carbene into the Os-Cl bond in **43** with a subsequent rearrangement to *η*^5^ coordination would furnish Cp complex **41**.

## 3. Reactivity of Five-Membered Metallapentalynes

Recently, Xia and coworkers documented a series of five-membered cyclic metal carbyne complexes, i.e., osmapentalynes [47,48,49,50,51] and ruthenapentalynes [52]. The carbine-carbon bond angles in the metallapentalynes are around 130°, which are much smaller than those of the acyclic metal carbynes (180°). The large ring strain associated with extreme distortion of the metal carbyne unit lead to the high reactivity of the metal–carbon triple bond, which exhibits a unique performance.

### 3.1. Formation of Osmapentalyne-Coinage Metal Complexes

**Scheme 10:**
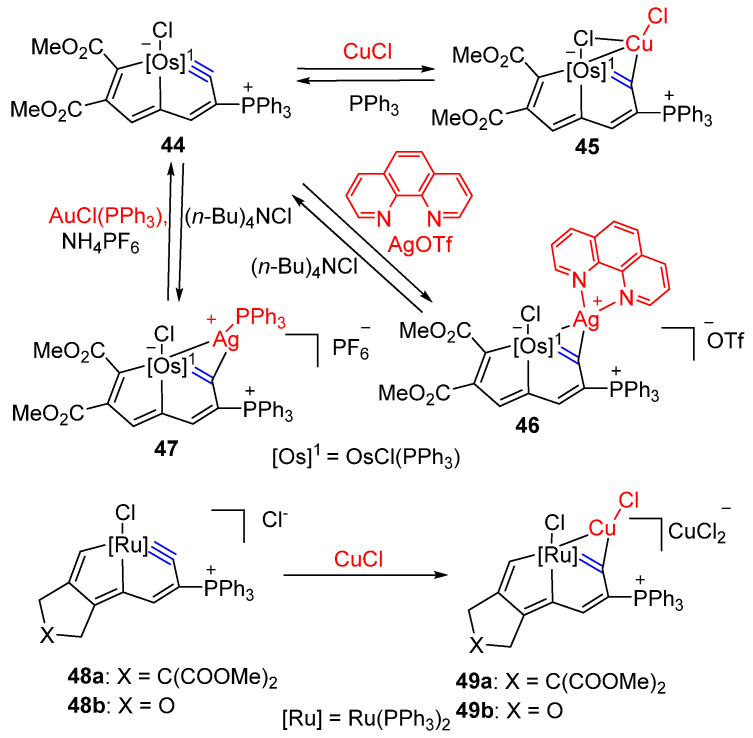
Reactions of metallapentalynes with coinage metal complexes.

The metal-carbon triple bond possesses an “alkyne-like” character and can react with an external metal precursor to generate bimetallic adducts, in which osmium-carbon triple bond coordinated with the coinage metal center. Xia reported that osmapentalyne **44 [48]** was treated with cuprous chloride to afford hetero bimetallic adduct **45**, in which the chloride bonded to osmium also coordinated with copper atoms. As different silver or gold precursors were subjected to osmapentalyne **44**, the corresponding hetero bimetallic adduct **46** or **47** was formed in a similar way. The interaction between cyclic osmacarbyne with coinage metal is weak, which is supported by the fact that in the presence of ligands (PPh_3_ for **45** or (*n*-Bu)_4_NCl for **46**–**47**) the bimetallic complex readily dissociates and osmapentalyne **44** would be regenerated (Scheme 10) [53]. Ruthenapentalynes **48a**–**b** readily reacted with CuCl to afford similar bimetallic complexes **49a** and **49b**, except that the chloride bonded to ruthenium did not coordinate with copper [52] 

### 3.2. Electrophilic Reaction

Alkynes, which exhibit electrophilic properties, would generate alkenes upon treatment of electrophiles. Carbynes and metallapentalynes are both sensitive to acids. The acyclic carbynes might generally form carbenes, while the shift of metal–carbon triple bond would be observed in metallapentalynes. In most cases, the fused rings of metallapentalynes would be reserved, which might be attributed to the higher stability derived from the aromaticity.

**Scheme 11:**
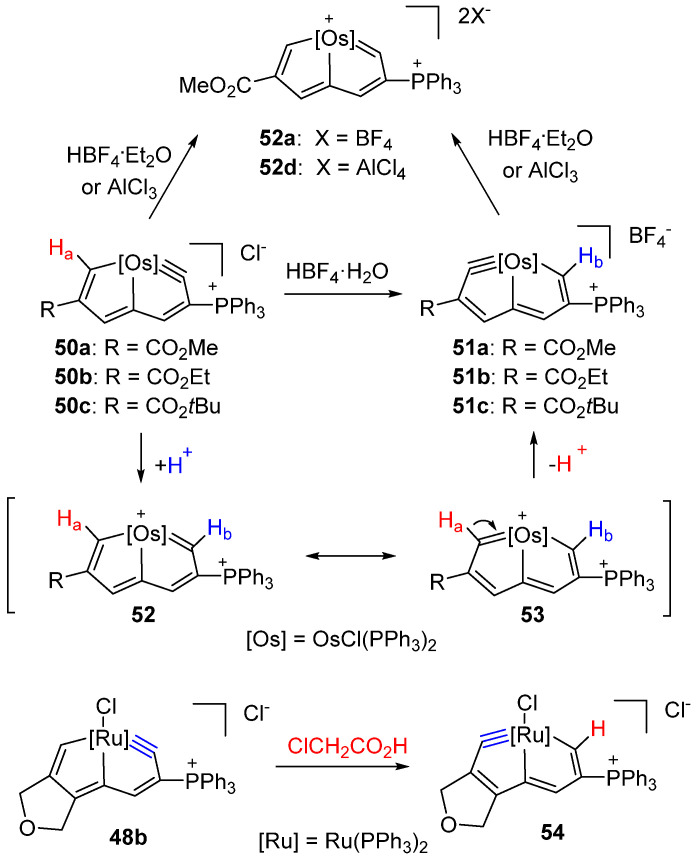
Metal-carbon triple bond shift of metallapentalynes.

Upon treatment of osmapentalynes **50a**–**c** [47] with HBF_4_·H_2_O at room temperature, the metal–carbon triple bond could shift from one ring to another to generate osmapentalynes **51a**–**c** [54]. The plausible mechanism was that the osmapentalyne was protonated to give the osmapentalene intermediate **52** bearing a 16-electron osmium center, which was not stable enough according to the 18-electron transition metal rule. Subsequent elimination of H_a_ would furnish osmapentalynes **51a**–**c** with an 18-electron osmium center. Fortunately, the 16-electron osmapentalenes, complex **52a** and **52d**, were captured and characterized by the reaction of osmapentalyne **50** or **51** with HBF_4_·Et_2_O or AlCl_3_ (Scheme 11). The metal carbyne bond shift reaction of the ruthenapentalyne **48b** is similar to that of osmapentalynes, as is the mechanism [52].

**Scheme 12:**
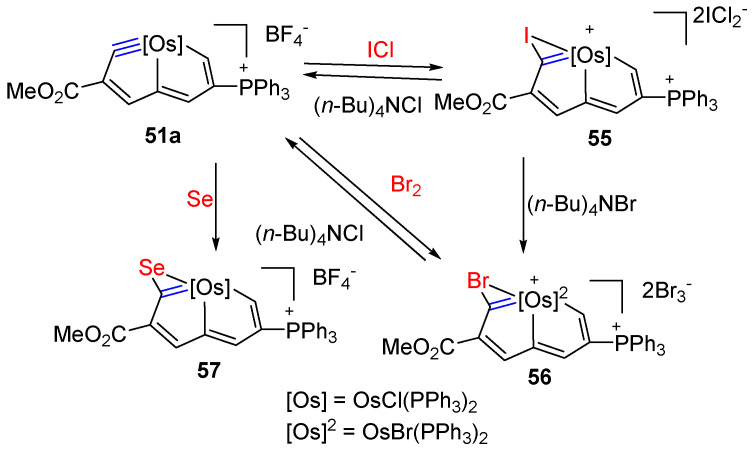
Halogenation of metallapentalynes.

The halogenation of metallapentalynes [55] would further demonstrate the electrophilicity of metallapentalynes. Treatment of osmapentalyne **51a** [47] with ICl or elemental Br_2_ led to the formation of the corresponding halogenated osmapentalene **55** or **56**, respectively, which could be viewed as the first examples of metallaiodirenium and metallabromirenium ions (Scheme 12). The well-characterized bromocarbene complex **56** or iodocarbene complex **55** are similar to the generally proposed intermediates in the halogenation of alkynes, which would further demonstrate the “alkyne-like” properties of metal-carbon triple bond in metallapentalynes. The nucleophilic substitution reaction of **55** with Bu_4_NBr would generate brominated complex **56**. However, the application of Bu_4_NCl to the halogenated osmapentalenes **55**–**56** would result in the elimination of the halogen cation and the regeneration of osmapentalyne **51a**. Moreover, **51a** also reacted with elemental selenium at 60 °C, leading to the formation of Se-containing osmapentalene **57** [56], which was regarded as the first example of σ-aromaticity dominating in an unsaturated Se-containing ring.

**Scheme 13:**
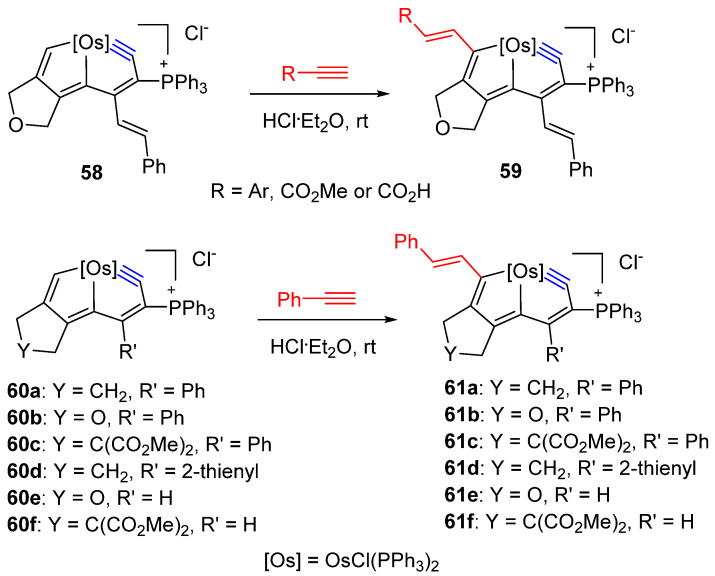
Reactions of alkynes with osmapentalynes.

The reactions of metal carbynes and carbon–carbon triple bonds tended to provide cycloaddition intermediates or products. In contrast, Xia and coworkers documented a fantastic reaction between osmapentalynes and terminal alkynes in 2020, giving rise to acyclic addition products **59** and **61** (Scheme 13) [57]. High efficiency, regio- and stereoselectivity were observed, furnishing exclusively *trans* and *anti*-Markovnikov products in excellent yields. These reactions possess good functional tolerance for both alkynes and osmapentalynes and are easy to handle (air atmosphere and ambient conditions). The plausible mechanism is the electrophilic addition of cyclic carbyne by the terminal alkynes under the synergistic effect of protons on the basis of experimental observations as well as theoretical calculations. The application of the reactions provides an easy access to functionalized d_π_-p_π_ conjugated systems, which exhibit great significance in functional materials.

### 3.3. Nucleophilic Reaction

**Scheme 14:**
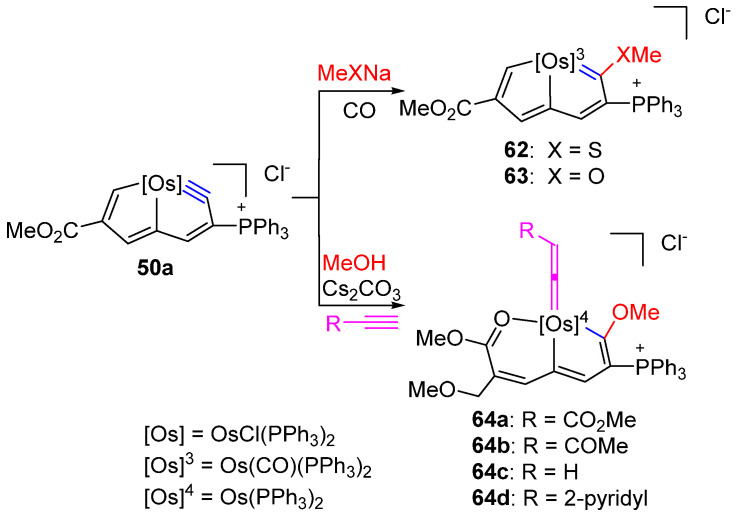
Nucleophilic reactions of osmapentalyne to form **62**–**64**.

The nonlinear distortion of the carbyne carbon angle of the osmapentalyne also facilitates the nucleophilic reaction. In contrast to the 16-electron osmapentalene **52** secured by protonation [54], 18-electron osmapentalenes **62** and **63** were achieved by the nucleophilic attack of the methanethioxide (MeS^−^) and the methanoxide (MeO^−^) anions towards the carbyne carbon of osmapentalyne **50a** under the atmosphere of carbon monoxide. [54] Xia and coworkers reported the transformation of osmapentalyne to osmafulvenallene **64** (Scheme 14), which constituted the pioneering example of well-characterized metallafulvenallenes [58].

**Scheme 15:**
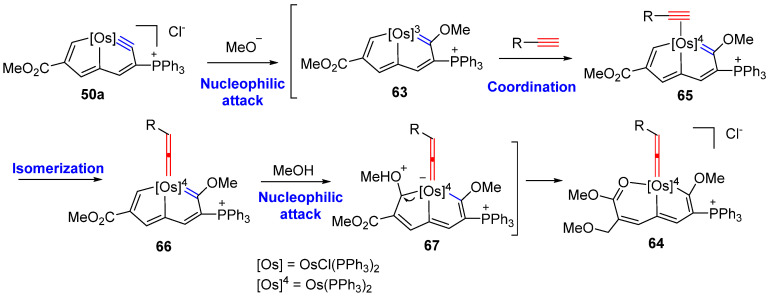
Proposed mechanism for the formation of **64**.

The rationale of the mechanism is depicted in Scheme 15. The combination of cesium carbonate and methanol would generate methanoxide, which conducted the first nucleophilic addition to a carbyne carbon, affording the metallapentalene intermediate **63**. Subsequent coordination with a terminal alkyne led to the π-alkyne complex **65**, which would form the vinylidene η^1^-complex **66** via isomerization. The α-carbon within the five-membered metallacycle was attacked by the second methanol molecule. Sequential ring opening, protonation and coordination of the ester group furnished the final fused metallafulvenallene complex **64**.

**Scheme 16:**
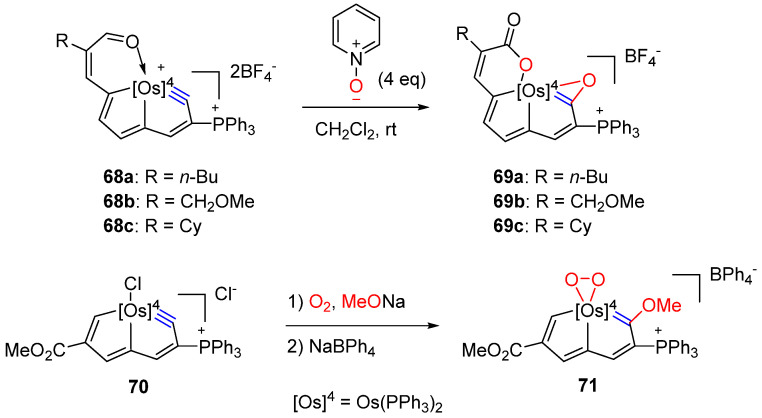
Oxygenation of osmapentalynes.

Very recently, oxygenation of osmapentalynes was reported by Xia’s group (Scheme 16). The first example was the oxygenation of the osmium carbolong complex ring [59]. Applying pyridine *N*-oxide as the oxidant, oxygen atoms were transferred to the metal–carbon triple bond in a nucleophilic reaction. Meanwhile, the labile aldehyde was oxidized to carboxylic acid, affording unprecedented OCCCO-type pentadentate chelates **69**. The second example was the direct oxygenation of the metal center in the carbolong complex **70** [60]. The mixture of osmapentalyne **70** and excess sodium methoxide under oxygen atmosphere resulted in almost quantitative formation of osmapentalene **71**, in which the dioxygen was bound to the metal center. The plausible mechanism was depicted as the nucleophilic attack at carbyne carbon atom by sodium methoxide, which led to the reduction of metal center and dissociation of chloride, which facilitated the coordination and the following reduction of dioxygen.

**Scheme 17:**
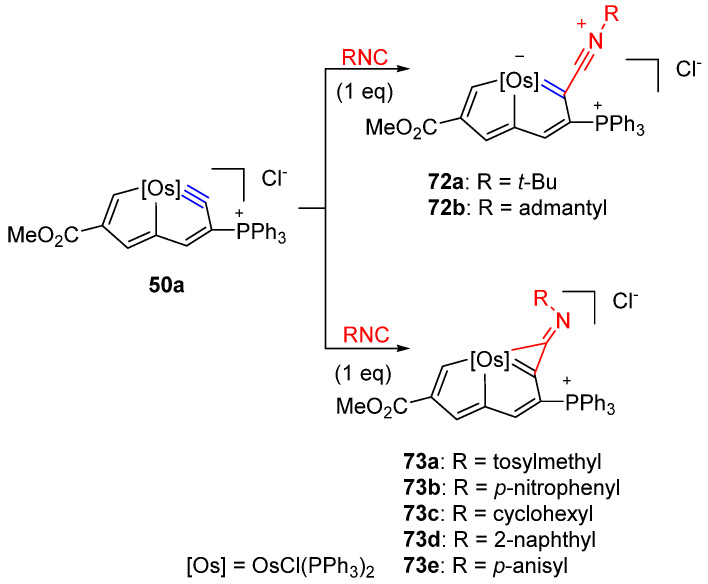
Nucleophilic reactions of **50a** with isocyanides.

The direct attack of a free isocyanide on the carbyne carbon atom of the osmapentalyne **50a** would lead to the formation of η^2^-iminoketenyl metallapentalene intermediates as well as the unprecedented metallaindene derivatives (Scheme 17). The steric hindrance of the bulky *N*-substituents *tert*-butyl or 1-adamantyl inhibited the bending at the isocyanide nitrogen, which prevented the formation of a η^2^-iminoketenyl structure and led to the osmapentalene **72a**–**b**. In comparison to bulky isocyanides, other less bulky isocyanides could afford the desired metallacyclopropenimine complex **73a**–**e**, which were often regarded as the intermediates in nucleophile-induced carbyne-isocyanide coupling processes. The intermediates with η^2^-iminoketenyl ligands had not been isolated and characterized until the η^2^-iminoketenyl metallapentalenes were documented by Xia and coworkers [61].

**Scheme 18:**
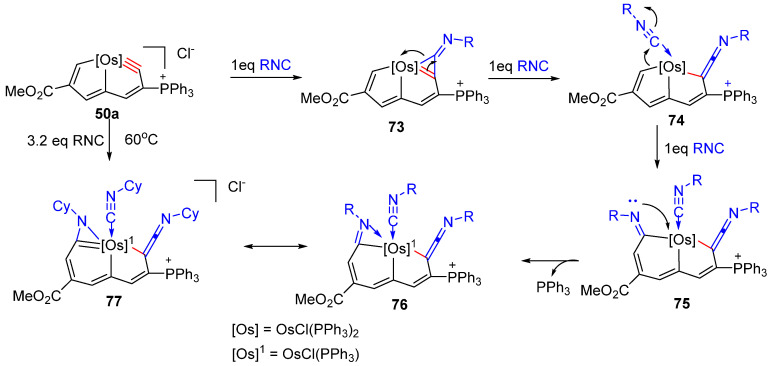
Nucleophilic reactions of **50a** to form metal-bridged metallaindene.

It is noteworthy that the addition of excess isocyanides at elevated temperature led to metallaindene derivatives, which represented the first metal-bridged polycyclic metallaaromatics [61]. Treatment of metallapentalyne **50a** with the first isocyanide provided η^2^-iminoketenyl metallapentalene **73**. The second isocyanide would coordinate with the osmium center to afford intermediate **74**, which underwent sequential isocyanide insertion and coordination of a third isocyanide to generate intermediate **75**. The exocyclic imine group could kick out one triphenylphosphine and coordinate with the osmium center, followed by subsequent aromatization to furnish metallaindene **77** (Scheme 18). The same research group extended the metallapentalene and metallaindene system to ruthenium except that the η^2^-iminoketenyl species were not observed [62].

**Scheme 19:**
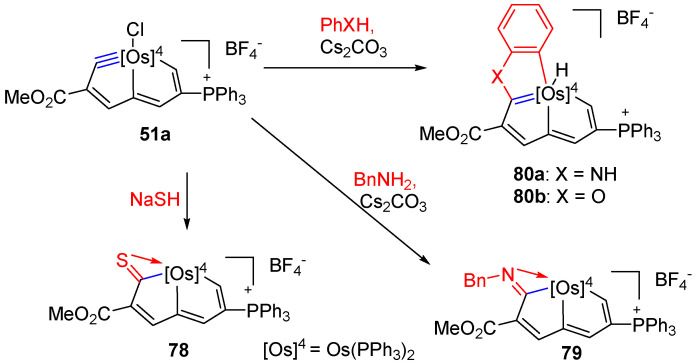
Nucleophilic reactions of osmapentalyne **51a** to form **78**–**80**.

The metal-carbon triple bond moiety in carbyne shifted complex **51a** is also sensitive to nucleophilic attack (Scheme 19). Thus, products **78** and **79** were formed by treating osmapentalyne **51a** with sodium hydrosulfide and benzylamine, respectively [47]. Noteworthy is that in the presence of aniline and cesium carbonate, osmapentalyne **51a** was transformed to the novel metal-bridged polycyclic aromatic system **80**, in which the metal center is shared by three aromatic five-membered rings [63]. The reaction involved the nucleophilic addition of aniline to the carbyne moiety and subsequent C-H bond activation (oxidative addition), providing complex **80a**. Treatment of osmapentalyne **51a** and phenol produced the similar product **80b** via a paralleled mechanism. A number of metal-bridged polycyclic aromatics, including different substitutions on the aromatic ring or larger fused-ring systems, were achieved with the same synthetic strategy [63,64,65].

**Scheme 20:**
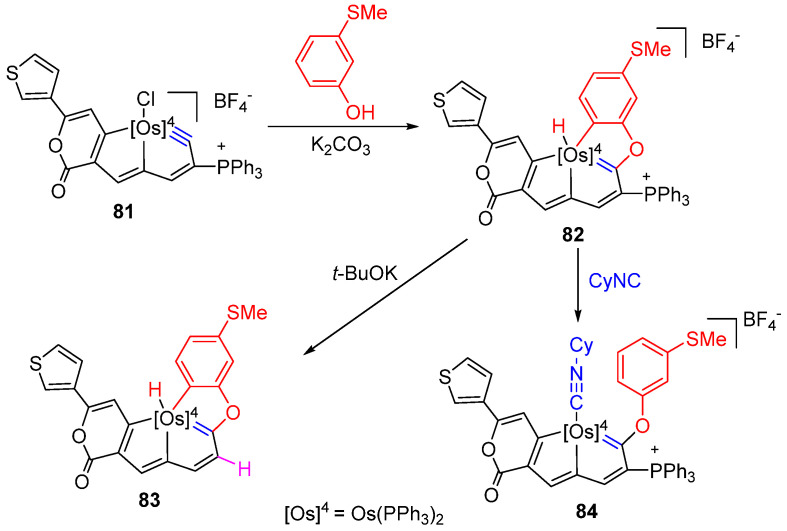
Nucleophilic reactions of osmapentalyne **81** and further transformation.

In the presence of arene nucleophile X, the lactone-fused osmapentalyne **81** [66] was converted to metal bridgehead polycyclic π conjugate systems **82**. Further transformations with *t*-BuOK or cyclohexyl isocyanide would lead to the neutral complex **83** or coordinated complex **84**, respectively (Scheme 20). The delocalization of the above mentioned three metalla-aromatics could be used to switch the charge transport pathway of single-molecule junctions and thus tune the charge transport abilities significantly [67], which shed light on the potential applications of metal-bridged polycyclic aromatics in materials science.

**Scheme 21:**
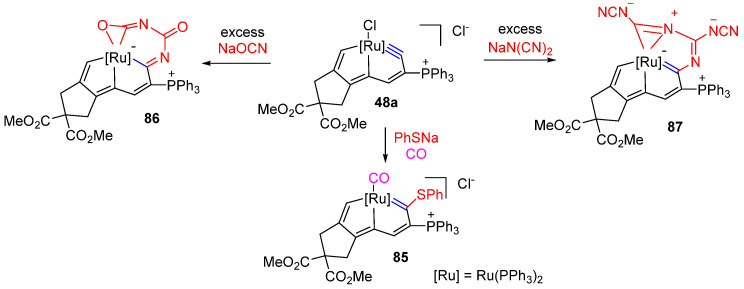
Nucleophilic reactions of ruthenapentalyne **43**.

The ruthenium-carbon triple bond moiety in ruthenapentalynes showed nucleophilic reactivity. The reaction of ruthenapentalyne **48a** with sodium thiophenoxide under carbon monoxide atmosphere resulted in the generation of the nucleophilic addition product ruthenapentalene **85**. On the basis of the rich and unique reactivity of ruthenapentalynes, Xia and coworkers designed and carried out fantastic cascade cyclization reactions with bidentate nucleophiles (sodium cyanate or sodium dicyanamide) to afford annulation complexes **86** or **87**, respectively (Scheme 21) [52].

**Scheme 22:**
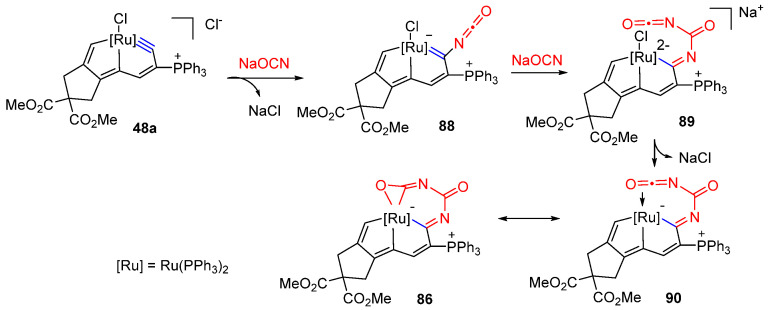
Proposed mechanism for the formation of **86**.

A plausible cascade cyclization mechanism is depicted in Scheme 22 [52]. The first cyanate ion initiated the nucleophilic attack at the carbyne carbon of **48a**, leading to the formation of ruthenapentalene intermediate **88**, which could be further attacked by the second cyanate ion to give intermediate **89**. The Cl ligand dissociates from intermediate **89** to form the intermediate **90**, which contains a vacant site at the ruthenium center. The subsequent coordination of the OCN group with the metal center could result in the final polycyclic complex **86**. The mechanism to access the annulation product **87** might parallel that of the above cascade cyclization reaction, additionally, the nucleophilic attack was initiated by the N(CN)_2_ ion.

### 3.4. Cycloaddition Reactions

The metallapentalynes exhibit rich metal carbyne reactivities due to the extreme strain in the fused five-membered ring containing a metal carbyne bond. The driving force for the cycloaddition reaction of metallapentalynes with the alkynes could be attributed to the release of the large ring strain in the five-membered ring of metallapentalynes. Xia and coworkers documented a number of cycloaddition reactions involving the metal-carbon triple bond in metallapentalynes [52,66,68,69,70,71,72,73].

**Scheme 23:**
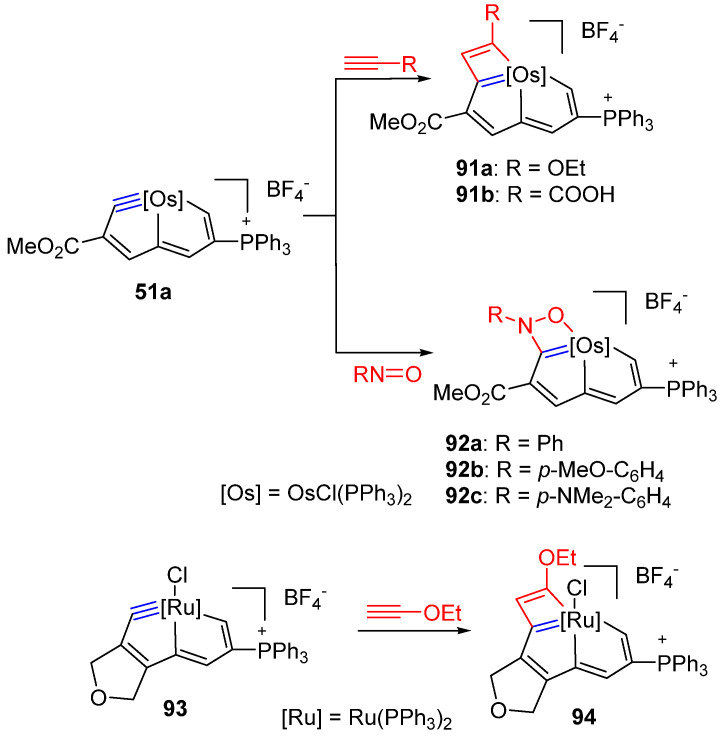
The [2+2] cycloaddition reactions of metallapentalynes with alkynes and nitrosoarenes.

Treatment of the cationic osmapentalyne **51a** with propionic acid or ethoxyacetylene provided the planar products **91a** and **91b**, respectively, through unprecedented [2+2] cycloaddition reactions of alkynes with a late-transition-metal carbyne complex [68]. It is noteworthy that two classical anti-aromatic frameworks, cyclobutadiene and pentalene, were stabilized by incorporating one transition metal fragment into the anti-aromatic systems, thus providing novel aromatic metallacycles. Complexes **91a** and **91b** constituted the first example of [2+2] cycloaddition products formed between a late-transition-metal carbyne with alkynes. Further inserti on was not observed in the presence of excess alkynes, thus preventing the formation of larger metallacycles. The reaction could be extended to early-transition-metal carbyne complexes. Treatment of ruthenapentalyne **93** with ethoxyacetylene furnished complex **94**, which constitute the first [2+2] cycloaddition reaction of ruthenium carbyne complex with an alkyne [52]. The first [2+2] cycloaddition reaction of metallapentalyne **51a** with nitrosoarenes (Ar–N=O) was documented by Xia et al. [69], affording well-characterized metallapentalenoxazetes **92a**–**c** (Scheme 23). Faster reaction rate was observed provided that the electron-donating substituents were introduced to the *para*-position of the nitrosophenyl ring (**92b**–**c**), which could be attributed to the enhanced nucleophilicity of the nitrogen atom.

**Scheme 24:**
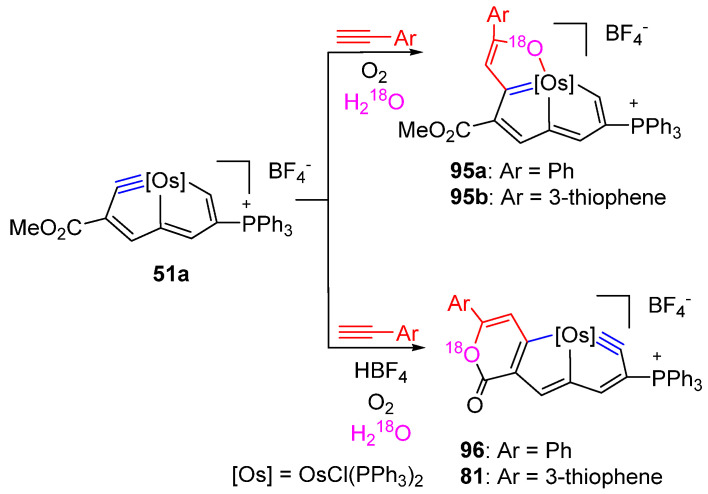
Synthesis of complexes **81**, **95**–**96**.

The [2+2] cycloadditions of osmapentalynes with alkynes were further investigated with different reaction conditions. In the presence of water and oxygen, osmapentalyne and arylacetylene were transformed to the first α-metallapentalenofuran **95**. Furthermore, ^18^O labeling experiments suggest that the oxygen atom in the furan ring comes from water [66]. With the synthetic methodology in hand, modifications were carried out to install olefinic groups in the metallapentalenofurans, which were then subjected to polymerization to provide metallapolymers exhibiting excellent stimuli-responsive properties [74,75]. The lactone-fused metallapentalynes **81** and **96** were formed if tetrafluoroboric acid was also present. Interestingly, the metal–carbon triple bond in osmapentalynes **51a** was shifted to another five-membered ring in complex **81** and **96** accompanied by the formation of new lactone ring (Scheme 24).

**Scheme 25:**
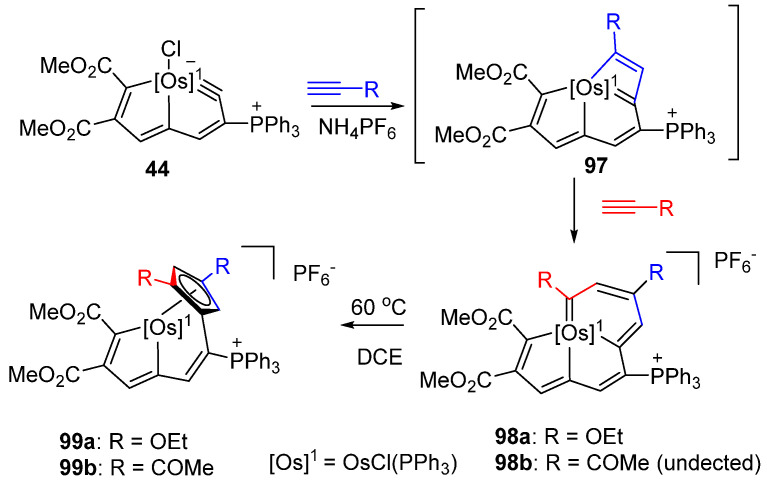
The [2+2+2] cycloaddition of metallapentalyne **44** with alkynes.

In 2018, Xia and coworkers advanced the above [2+2] cycloaddition to establish the first example of a [2+2+2] cycloaddition reaction of an alkyne with a late-transition-metal carbyne complex [48]. Upon treatment of NH_4_PF_6_, neutral osmapentalyne **44** underwent [2+2] cycloaddition with the first alkyne. Meanwhile, the chloride ligand of the osmium center was kicked out to afford a cationic intermediate **97**, which exhibited potent reactivity to be inserted by the second alkyne, affording non-planar eleven-carbon framework **98**. Subsequent migratory insertion furnished the thermally stable η^5^-Cp complex **99** (Scheme 25).

**Scheme 26:**
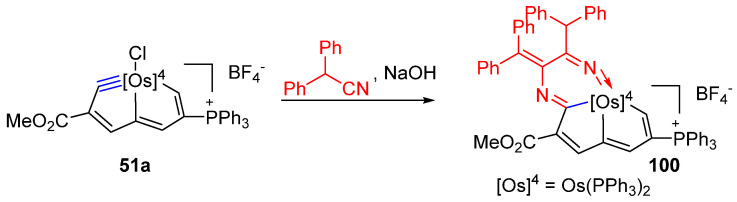
The [2+2+2] cycloaddition of metallapentalyne **51a** with nitriles.

In 2019, the same research group reported the formal [2+2+2] cycloaddition reaction of a metal-carbyne triple bond with nitriles, demonstrating the “alkyne-like” properties of metal–carbon triple bonds. Treatment of osmapentalyne **51a** with 2,2-diphenylacetonitrile in the presence of sodium hydroxide resulted in the formation of tricyclic metallapyrazine **100** (Scheme 26) [70].

**Scheme 27:**
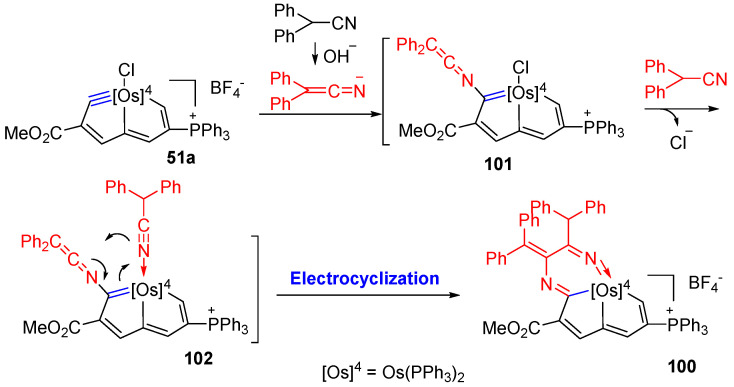
Mechanism of [2+2+2] cycloaddition of metallapentalyne **51a** with nitriles.

The reaction mechanism was rationalized as follows (Scheme 27). The deprotonation of 2,2-diphenylacetonitrile with sodium hydroxide provided the ketenimine intermediate as a nucleophile, which could attack the carbyne carbon to give intermediate **101**. The chloride ligand was kicked out and replaced with second molecule of 2,2-diphenylacetonitrile to afford intermediate **102**, which would facilitate the 6π electrocyclization reaction to furnish metallapyrazine complex **100**.

**Scheme 28:**
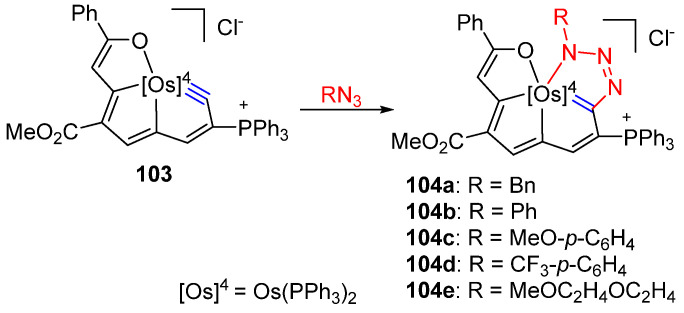
Metalla-click reactions.

Very recently, Xia’s group enriched the “alkyne like” reactivity of metal–carbon triple bond. Osmapentalyne **103** was prepared from the deprotonation of metallapentalene, furnishing the first cyclic metal carbyne in tricyclic metalla-aromatics with a bridgehead metal, which was subjected to a range of diversely functionalized azides. Excellent yields, regioselectivity and compatibility were observed to provide an unprecedented series of polycyclic aromatics (**104a**–**e**), in which a bridgehead metal center was shared by four five-membered aromatic rings, thus representing a typical “metalla-click” reaction (Scheme 28) [71]. The construction of polycyclic aromatics that share a bridgehead atom with more than three rings has never been accomplished before this work, which therefore broke the record.

**Scheme 29:**
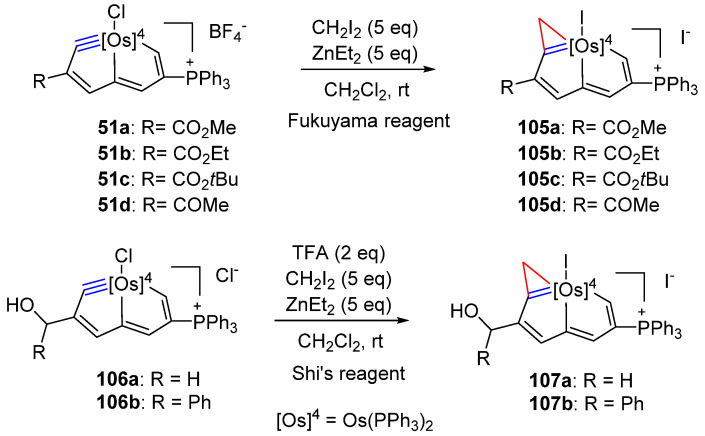
Simmons-Smith reactions.

During the preparation of this manuscript, Xia and coworkers secured the Simmons-Smith reactions of substrates with metal–carbon triple bonds for the first time [72] (Scheme 29). Osmapentalyne **51a** was transformed into cyclopropenation product **105a** in the presence of classical Fukuyama reagent. The substrate scope was examined with electron-withdrawing substituents to give the expected cyclopropene products **105b**–**d** in good yields. When the substituents were switched to carbinols, the Fukuyama reagent failed, and the more reactive Shi’s reagent was used to furnish the desired product **107**. These results demonstrate that the reactions were sensitive to electronic effect, which was substantiated by theoretical calculations. Based on the results achieved, the Simmons–Smith reactions were also extended to ruthenapentalynes [72].

**Scheme 30:**
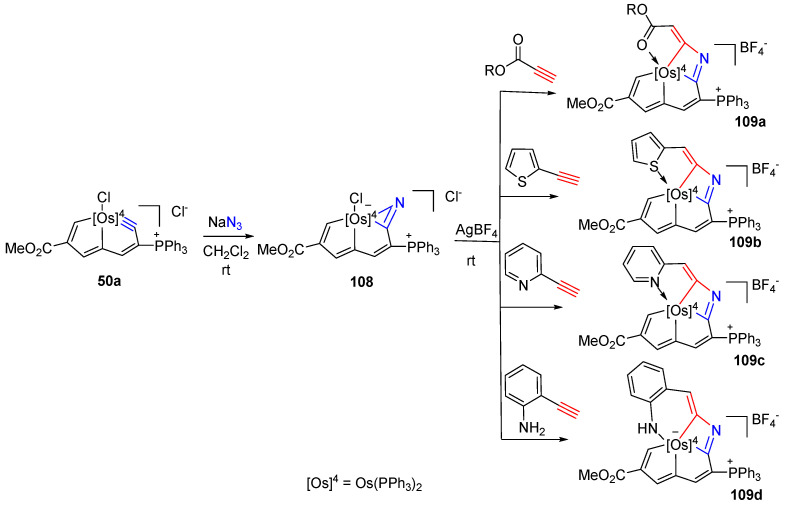
Formal [3+1] cycloadditions.

Unprecedented [3+1] cycloadditions of metalla-azirines with terminal alkynes were documented by Xia’s [73] group (Scheme 30). Osmapentalyne **50a** was transformed into the metalla-azirine **108** in the presence of sodium azide. The reaction was the first synthesis of a metalla-azirine by [2+1] cycloaddition reaction of a metal carbyne with an azide and paralleled the first [3+2] cycloadditions of late-transition-metal carbyne with organic azides [71]. In the presence of AgBF_4_, a series of substituted terminal alkynes as well as metalla-azirine **108** were transformed to various tetracyclic complexes **109a**–**d**. Moreover, the five coordinating atoms lie in the equatorial plane in the CCCCX-type (X = N, O, S) carbolong complexes obtained and the reaction provides a valuable supplement to the construction of planar pentadentate chelates.

## 4. Conclusions

Fantastic molecular architectures characterized with aromaticity have drawn considerable attention from the synthetic community, thus producing a huge number of aromatic structures with elegance as well as variety. As commonly sensed, the organic alkyne exhibits a linear shape and the bond angles around the acetylenic carbons are 180°. Many efforts have been devoted to the chemistry of cyclic complexes containing metal-carbon triple bonds, in which the carbine-carbon bond angles were less than 180° since the first example of metallabenzyne in 2001. Cyclic complexes containing carbynes could be classified into six-membered metallacarbynes and five-membered metallapentalynes. The carbine-carbon bond angles of metallabenzynes are around 148°. Such a record was held for 12 years until the discovery of metallapentalynes, whose carbine-carbon bond angles were bent to around 130°, far away from the ideal value of 180°. Such extreme distortion results in considerable large ring strain, resulting in the unprecedented high reactivity.

The reaction types of metallabenzynes could be classified into four types, such as ligand exchange reactions, electrophilic reactions, nucleophilic reactions and migratory insertion reactions. Ligand exchange reactions are the fundamental reactions in organometallic chemistry and were unsurprisingly involved in the reactions of metallabenzynes and metallapentalynes. Due to the ambiphilic properties of carbynes, both electrophilic and nucleophilic reactions were observed in the reactions of metallabenzynes and metallapentalynes, which resemble the classical metal carbyne complex. Metallabenzynes exhibited excellent aromatic properties and the whole cyclic system acted as an entire entity under common electrophilic conditions, thus resembling the electrophilic substitution reactions displayed upon organic aromatics. The carbyne moiety stayed intact, whereas the aromatic rings were attacked by electrophiles. One exception that was observed was that the carbyne moiety was epoxidized upon treatment of hydrogen peroxide. In most cases, the nucleophiles would attack the carbyne carbon. However, “softer” nucleophiles might attack the *para* position of the six-membered ring system, thus demonstrating the aromaticity of metallabenzynes. Meanwhile, the aromaticity of osmabenzynes was strongly affected by the corresponding substituents of metallacycles and ligands of metal center, leading to the decomposition of metallacarbyne ring systems to form cyclopentadiene complexes via migratory insertion reactions. In sharp contrast, the ring systems of metallapentalynes were never broken throughout all the reported reactions, which might be attributed to the higher stability of metallapentalynes derived from their strong aromaticity despite the extremely strained bond angles of metallapentalynes. The metal–carbon triple bond in metallapentalynes possessed “alkyne-like” character, which was demonstrated by the formation of osmapentalyne-coinage metal complexes and cycloaddition reactions.

Recent advances in the reactivity of a series of cyclic complexes containing carbynes described herein deepened our understanding of the nature of metallabenzynes and metallapentalynes and resulted in the production of a number of novel metallacycles. However, the research on the reactivity of metallabenzynes and metallapentalynes is still limited compared with that of organic alkynes and metal-carbon triple bonds. Additionally, the reactivity studies of these unique species need further exploration, and the isolation of other metalla-aromatics is also anticipated via the corresponding research. Considering that metallacycles are regarded as intermediates in many transition-metal-catalyzed reactions, this study provides a new perspective for the application of late-transition-metal carbyne species as catalysts.

The unique structure leads to novel properties. Novel metallacycles derived from the reactivity studies of metallapentalynes exhibited great application prospects in various areas, such as near-infrared dyes [47], photothermal therapy [74,75,76,77], phototherapy [78], and self-healing materials [79], enabling their potential applications in materials science and biomedicine. Future developments of metallabenzynes and metallapentalynes and exploration of their potential applications in catalysis [60], materials science and biomedicine would not only extend our perception of aromaticity in metallacycles, but also further enrich fundamental organometallic chemistry.
